# S-Nitrosoglutathione Reverts Dietary Sucrose-Induced Insulin Resistance

**DOI:** 10.3390/antiox9090870

**Published:** 2020-09-15

**Authors:** Inês Sousa-Lima, Ana B. Fernandes, Rita S. Patarrão, Young-Bum Kim, M. Paula Macedo

**Affiliations:** 1 CEDOC, NOVA Medical School/Faculdade de Ciências Médicas, Universidade Nova de Lisboa, 1150-090 Lisboa, Portugal; ines.lima@nms.unl.pt (I.S.-L.); ana.fernandes@neuro.fchampalimaud.org (A.B.F.); rita.patarrao@nms.unl.pt (R.S.P.); 2 Division of Endocrinology, Diabetes and Metabolism, Beth Israel Deaconess Medical Center and Harvard Medical School, Boston, MA 02115, USA; ykim2@bidmc.harvard.edu; 3 IGC—Instituto Gulbenkian de Ciência, 2780-156 Lisbon, Portugal; 4 APDP-Diabetes Portugal, Education and Research Center, 1250-203 Lisbon, Portugal; 5 Department of Medical Sciences, Institute of Biomedicine University of Aveiro, 3810-193 Aveiro, Portugal

**Keywords:** insulin resistance, glucose homeostasis, liver, nitric oxide, glutathione, S-nitrosoglutathione, insulin sensitivity

## Abstract

The liver is a fundamental organ to ensure whole-body homeostasis, allowing for a proper increase in insulin sensitivity from the fast to the postprandial status. Hepatic regulation of glucose metabolism is crucial and has been shown to be modulated by glutathione (GSH) and nitric oxide (NO). However, knowledge of the metabolic action of GSH and NO in glucose homeostasis remains incomplete. The current study was designed to test the hypothesis that treatment with S-nitrosoglutathione is sufficient to revert insulin resistance induced by a high-sucrose diet. Male Wistar rats were divided in a control or high-sucrose group. Insulin sensitivity was determined: (i) in the fast state; (ii) after a standardized test meal; (iii) after GSH + NO; and after (iv) S-nitrosoglutathione (GSNO) administration. The fasting glucose level was not different between the control and high-sucrose group. In the liver, the high-sucrose model shows increased NO and unchanged GSH levels. In control animals, insulin sensitivity increased after a meal or administration of GSH+NO/GSNO, but this was abrogated by sucrose feeding. GSNO was able to revert insulin resistance induced by sucrose feeding, in a dose-dependent manner, suggesting that they have an insulin-sensitizing effect in vivo. These effects are associated with an increased insulin receptor and Akt phosphorylation in muscle cells. Our findings demonstrate that GSNO promotes insulin sensitivity in a sucrose-induced insulin-resistant animal model and further implicates that this antioxidant molecule may act as a potential pharmacological tool for the treatment of insulin resistance in obesity and type 2 diabetes.

## 1. Introduction

The prevalence of diabetes, particularly type 2 diabetes (T2D), is increasing worldwide [1]. The development of T2D is now agreed to be a consequence of both lifestyle and genetic background [2,3]. Given that sugar intake has markedly increased in the last decades [4,5,6,7], it is now accepted that diet is a primary environmental factor for the onset of T2D. The consumption of refined carbohydrates has deleterious effects in the progression of insulin resistance and several reports show a correlation between exacerbated sugar intake and higher rates of obesity, diabetes, hypertension, and cardiovascular disease [8,9,10]. However, the detailed mechanism underlying diet-induced insulin resistance is not yet fully understood.

The sucrose present in the diet yields equal amounts of glucose and fructose. Rising amounts of glucose consumption are well documented as one of the major causes of insulin resistance [11]. Presently it is known that fructose intake is also a key risk factor for carbohydrate-induced insulin resistance [12,13]. Several studies have emerged in the last decade concerning insulin resistance associated to high sugar diets [14,15,16]; however, due to the variability on the time of exposure to the diet, percentage of the sugar within the diet, and initial and endpoint of the exposure, the metabolic alterations are inconsistent and lack any mechanism underlying the dysregulation observed in each case. Moreover, none of the articles showed any pharmacological approach to revert diet-induced insulin resistance.

Ribeiro et al. reported that insulin resistance induced by sucrose feeding is due to an impairment of the hepatic parasympathetic nervous system in rats [16]. This occurs early in the development of sucrose-induced insulin resistance and continues through time, up until 9 weeks of sucrose feeding. Moreover, sucrose diet, at a time when bodyweight, fasting glycemia, blood pressure, and direct metabolic effects of insulin are not yet compromised, showed consistently an entire loss of insulin action. This insulin resistant state is likely to be a consequence of a compromised hepatic parasympathetic nervous system, impairing physiological hepatic nitric oxide (NO) production [16].

Our body is routinely exposed to exogenous agents that promote reactive oxygen species’ production as free radicals that lead to oxidation of the cellular machinery. To counteract the deleterious effects of reactive oxygen species there are endogenous antioxidant systems or exogenous antioxidants in the diet that neutralize these species, thus ensuring whole-body homeostasis. Oxidative stress is therefore likely a consequence of the imbalance between reactive species and antioxidants and is present in several pathological conditions such as type 2 diabetes [17].

The fact that sucrose-fed animals have increased hepatic peroxinitrite and an impaired antioxidant system suggests that the former is involved on the onset of insulin resistance [18,19,20]. Guarino et al. also observed that pharmacological inhibition of both hepatic glutathione (GSH) and NO could be related to the genesis of decreased insulin action, proposing the importance of these molecules in regulating normal insulin action [21,22,23].

GSH and NO react in physiological conditions to form S-nitrosoglutathione (GSNO) [24,25]. The major outcome of this physiological reaction is the ability to stabilize and transport NO through the bloodstream; recently, this molecule has been discovered to have important antioxidant properties [26,27,28]. The use of GSNO as a nitric oxide donor seems to have important implications besides just the nitric oxide delivery [29,30,31]; in fact, GSNO have been shown to promote insulin sensitivity when given intravenously to fasted healthy animals, thus showing a role for this molecule in insulin action [32]. Under physiological conditions nitrosothiols are capable of protein nitrosylation impacting on GLUT4-dependent glucose uptake in insulin-sensitive myotubes [33]. Evidence demonstrated that a nitric oxide donor, S-nitroso-N-penicillamine (SNAP), leads to an increase in both GLUT4 mRNA and protein levels L6 myotubes, which is thought to be mediated via an AMPK-dependent mechanism [34].

A pharmacological approach, in which glutathione and nitric oxide or S-nitrosoglutathione are used, specifically in sucrose-induced insulin resistance, has not been evaluated. Therefore, we hypothesize that administration of both GSH+NO or GSNO, in an animal model of sucrose-induced insulin resistance, will restore systemic insulin sensitivity. Here we show, for the first time, that GSNO can rescue insulin resistance induced by chronic exposure to sucrose feeding and may be a potential target for the treatment of metabolic-related disorders such as type 2 diabetes. 

## 2. Materials and Methods

### 2.1. Presurgical Protocols

Male Wistar rats, from the animal house of the NOVA Medical School—Faculdade de Ciências Médicas, Lisbon, Portugal, were housed in a temperature-controlled room, in a 12 h light/dark cycle. Rats had ad libitum access to standard chow (Special Diets Services, Essex, UK). The animals were fasted for 24 h. Rats were anesthetized with an intraperitoneal (IP) injection of sodium pentobarbital (65 mg/kg) and anesthesia was maintained throughout the experiment by continuous infusion into the femoral vein (10 mg/h/kg). The temperature was maintained at 37.0 ± 0.5 °C using a heating pad (Homeothermic Blanket Control Unit 50-7061, Harvard Apparatus, MA 01746, USA) and monitored with a rectal probe thermometer. All the animals were treated according to the European Union Directive for Protection of Vertebrates Used for Experimental and other Scientific Ends (86/609/CEE).

### 2.2. Surgical Protocol

The trachea was catheterized (polyethylene tubing, PE 240, Becton Dickinson, Franklin Lakes, NJ 07417-1880, USA) to allow spontaneous respiration. Both the femoral artery and vein were catheterized (polyethylene tubing, PE 50, Becton Dickinson, Franklin Lakes, NJ 07417-1880, USA) to establish an arterial–venous shunt, primed with heparin 200 IU/kg and saline. Blood pressure was continuously monitored by a Powerlab 8/s, AD Instruments Ltd., Oxford, OX4 6HD, UK; Chart/Maclab Software, AD Instruments Ltd., Oxford, OX4 6HD, UK. By clamping the venous outlet of the arterial–venous shunt, mean arterial pressure (MAP) was determined. Rats stabilized from the surgical intervention for at least 30 min before any procedure. After the stabilization period, arterial blood samples (25 μL) were collected every 5 min and glucose concentration was determined, using a glucose analyzer (1500 YSI Sport, Yellow Springs Instruments, OH, USA). When three successive stable glucose values were obtained a basal glucose level was determined.

### 2.3. Rapid Insulin Sensitivity Test (RIST)

The methodology chosen to evaluate insulin sensitivity was the previously described rapid insulin sensitivity test (RIST) [35]. The RIST started with the administration of an intravenous (IV) insulin bolus (50 mU/kg), over a period of 5 min, using an infusion pump (Perfusor fm, BBraun, Portugal). One minute after starting the insulin administration, glucose infusion (D-Glucose, 100 mg/mL, IV) was started at a rate of 3 mg/kg/min, to avoid hypoglycemia. Arterial glucose levels were measured at 2 min intervals and the rate of glucose infusion was adjusted, in order to maintain the initial baseline level. When no further glucose was required, the test was concluded. 

### 2.4. Determination of Serum Levels of Glucose, Insulin, and c-peptide

Glycemia was determined by the glucose oxidase method, using a glucose analyzer (1500 YSI Sport, Yellow Springs Instruments, OH, USA). Sera levels of insulin and c-peptide were determined by proper ELISA Kits (Mercodia, Uppsala, Sweden). 

### 2.5. Meal Tolerance Test

For the meal tolerance test a commercially available meal from Nestlé, Boost^®^, 1800 Vevey, Switzerland, was administered intraenterically at a dose of 10 mL/kg of body weight. The meal had 1.0 kcal/mL and its caloric distribution was: protein—17% (42 mg/mL); carbohydrate—68% (173 mg/mL); and fat—15% (16.9 mg/mL). After meal administration blood glucose levels were monitored at 0, 2, 5, 10, 20, 30, 45, 60, 90, and 120 min.

### 2.6. Measurement of Hepatic Nitric Oxide Synthase (NOS) Gene Expression

Hepatic nitric oxide synthase (NOS) gene expression was determined in control and sucrose-diet animals. The NOS expression was determined by real-time reverse transcriptase polymerase chain reaction (rtRT-PCR). All the NOS isoforms (endothelial (eNOS), neuronal (nNOS), and inducible (iNOS)) were determined. Liver total RNA was isolated using the GenElute Mammalian Total RNA Kit (Sigma Aldrich, St. Louis, MO, USA). Total RNA was reverse transcribed into cDNA, using the random-primers-based High Capacity cDNA Archive Kit (Applied Biosystems by Thermo Fisher Scientific, Waltham, MA, USA). Reverse transcriptase-polymerase chain reaction (RT-PCR) in real time was performed using Taqman probes methodology, as described previously [36]. For each primer/probe set, the detected reference sequences and the location on the gene were the following: NOS 1 (neuronal), Rn00583793_m1; NOS 2 (inducible), Rn0056146; and NOS 3 (endothelial) Rn02132634_s1 (Applied Biosystems by Thermo Fisher Scientific, Waltham, MA, USA). The results are expressed as relative quantification (RQ%) relative to the endogenous control (β-actin) and fasted control animals (100% expression).

### 2.7. Measurement of Hepatic Nitric Oxide Synthase (NOS) Activity

Total NOS activity was assessed by conversion of radiolabeled arginine to citruline ([3H]citruline), as previously described [37], using a NOS Activity Assay Kit (Cayman Chemical Company, Ann Harbor, MI, USA). Protein content was determined by the Bradford method [38]. 

### 2.8. Nitric Oxide Assessment

Liver NO levels were assessed by chemiluminescence-based measurement of nitrate (NO^3−^) and nitrite (NO^2−^) concentrations, as previously described [39]. Although nitrate is the major metabolite of NO, both species were used to estimate NO concentration in the liver [40,41,42,43]. This method consists in the reduction of both NO^2−^ and NO^3−^ to NO, at high temperature (90 °C), using vanadium III as the reducing agent; NO detection was made using a Sievers 280 NO Analyzer (Sievers Instruments).

### 2.9. Hepatic Glutathione Measurement

Hepatic glutathione was determined by the measurement of the absorbance at 414 nm using a colorimetric kit (Oxis Research™, Horsham, PA 19044, USA). This method uses an enzymatic recycling method for the quantification of total glutathione as previously described [44].

### 2.10. Cell Culture for and L6 Myotubes

L6 myotubes (kindly provided from Amira Klip, Hospital for Sick Children, Toronto, ON, Canada) were maintained as described [45].

### 2.11. Glucose Uptake

Glucose transport was measured as described previously. Briefly, L6 myotubes were serum starved for 3 h and then treated with 0–100 nM insulin, GSNO or GSNO + insulin or a vehicle for 30 min at 37 °C. [3H]-2-deoxy-D-glucose was added to the medium, cells were incubated for 10 min, and the level of [3H]-2-deoxy-D-glucose uptake into cells was expressed as a percentage of basal glucose transport in control cells [46].

### 2.12. Immunoblotting Analysis

Cell lysate proteins (20–50 g) were resolved by SDS-PAGE and transferred to nitrocellulose membranes. The membranes were incubated with polyclonal antibodies against phospho-Thr308 Akt (Cell Signalling); phospho-Tyr1158/1162/1163 IR (Merck Millipore, Burlington, MA, USA); IR (Santa Cruz Biotechnology, Inc., Dallas, TX, USA); Akt (Santa Cruz Biotechnology, Inc., Dallas, TX, USA); and actin (Santa Cruz Biotechnology, Inc., Dallas, TX, USA). The bands were visualized with enhanced chemiluminescence and quantified by densitometry [47].

### 2.13. Experimental Protocols

All animals were tested at 12 weeks of age and divided in two groups. The standard diet had access to normal water during the 12 weeks of the study. The study group had access to a 35% liquid sucrose (*w*/*v*) solution, for four weeks (from 8 to 12 weeks of age). Both groups had access to standard chow. Body weight and food and drink intake were monitored every 4 days after starting the sucrose diet.

At 12 weeks, insulin sensitivity was determined in all animals. Insulin sensitivity was assessed in both groups, control (*n* = 6) vs. sucrose (*n* = 4), after 24 h of fasting and after ingestion of a commercially available meal (Boost^®^ Nestlé, 10 mL/kg, 60 mL/h, intraenteric administration, 1800 Vevey, Switzerland). In a separate set of experiments insulin sensitivity was determined in both groups in the fasted state and after intraportal administration of a nitric oxide donor—50 μmol/kg of 3-morphosydnomine (SIN-1) and 1 mmol/kg of glutathione mono-esther (GSH-E; control: *n* = 10—fast vs. *n* = 9—GSH + SIN-1; sucrose: *n* = 4–7—fast vs. *n* = 3—GSH + SIN-1/*n* = 4 − 2× (GSH + SIN-1)). The doses were chosen according to the previous work performed by Guarino et al. Briefly both drugs were administered directly into the portal vein as a 10 min bolus. In the sucrose-fed group SIN-1 and GSH-E were administered at a higher dose (100 μmol/kg and 2 mmol/kg, respectively) to investigate a dose–response effect in the administration of both drugs.

In a third protocol GSNO was administered and insulin sensitivity determined either in the fast state and postdrug administration (control: *n* = 7—fast vs. *n* = 7 – GSNO; sucrose: *n* = 4–8—fast vs. *n* = 4—GSNO/*n* = 8—2× (GSNO)). GSNO was given at two different doses: 50 and 100 μmol/kg, the latter being given only to the sucrose-fed group. GSNO was administered intravenously as a 10 min bolus infusion.

To assess hepatic nitric oxide synthase activity and expression, as well as hepatic nitric oxide and glutathione levels, an independent set of experiments was performed where livers were harvested from control and sucrose fed rats. Hepatic NOS activity was determined in control (*n* = 10 fasted vs. *n* = 6 postprandial) and sucrose (*n* = 6 fasted vs. *n* = 7 postprandial) animals. Hepatic NOS expression was determined in control (*n* = 8) and sucrose (*n* = 5–11) animals. Hepatic NO was determined in control (*n* = 8 fasted vs. *n* = 9 postprandial) and sucrose (*n* = 3 fasted vs. *n* = 5 postprandial). Hepatic glutathione was determined in control (*n* = 14 fasted vs. *n* = 14 postprandial) and sucrose (*n* = 10 fasted vs. *n* = 8 postprandial). 

### 2.14. Statistical Analyses

Data are presented as means SEM. Areas under the curve (AUC) and linear regression analyses were done using GraphPad Prism 5.01 (GraphPad Software, La Jolla, USA). Differences significance was calculated through a two-tailed Student’s *t* test and a one-way ANOVA followed by Tukey–Kramer multiple comparison tests or repeated measures ANOVA, as applicable, using the GraphPad Prism 8.0.1 (San Diego, CA 92108, USA). Differences were accepted as statistically significant at *p* < 0.05.

## 3. Results

### 3.1. High Sucrose Intake Promotes Body Weight Gain with Unchanged Fasting Glycemia

**Figure 1:**
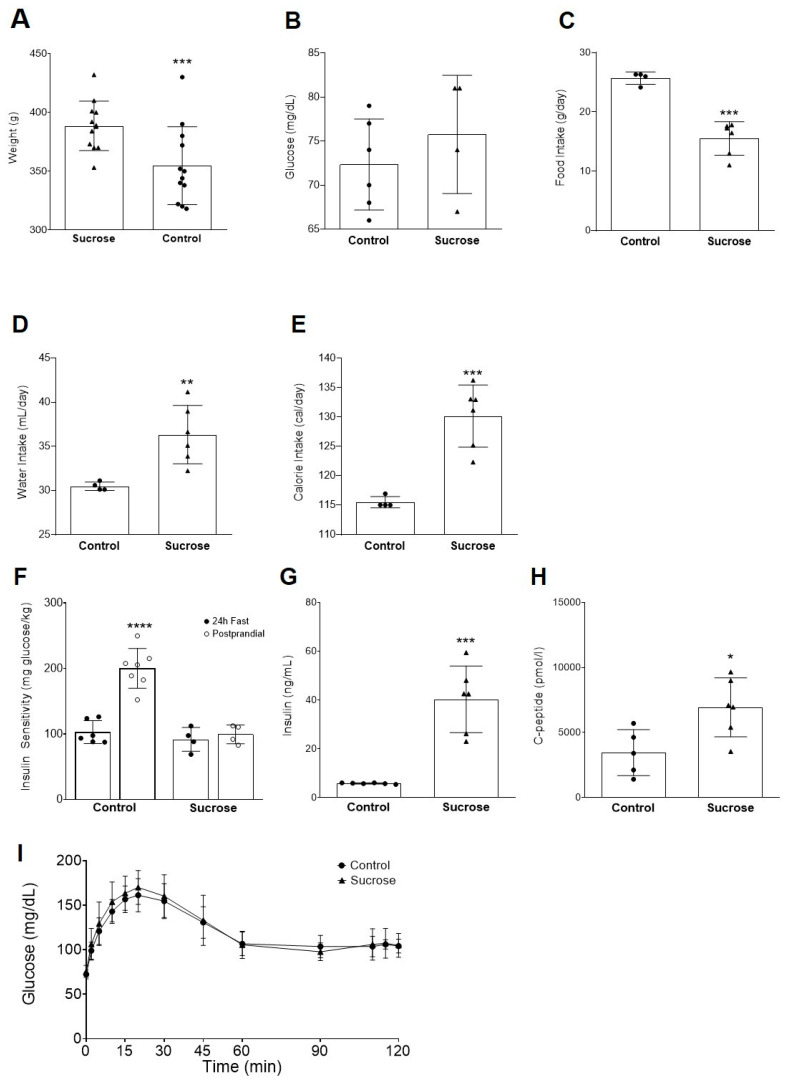
Effect of high-sucrose feeding on metabolic parameters, insulin sensitivity, and whole-body homeostasis in male Wistar rats. (**A**) Body weight; (**B**) fasting blood glucose; (**C**) food intake; (**D**) water intake; (**E**) calorie intake; (**F**) control and sucrose groups insulin sensitivity—fast and postprandial; (**G**) serum insulin levels; (**H**) serum C-peptide levels; and (**I**) meal tolerance test—MTT. Food, water, and calorie intake were measured throughout the study. All other parameters were determined at 12 weeks of age, blood glucose was measured after a 24 h fasting period, insulin and c-peptide were measured in random-fed. All rats were subjected to an MTT; after the fasting period rats were given a commercially available meal (Boost^®^ Nestlé) and blood was drawn from 0 to 120 min after meal administration for the measurement of glycemia. Data are presented as mean ± SEM for 4–12 rats per group. * *p* < 0.05; ** *p* < 0.01, *** *p* < 0.001, and **** *p* < 0.0001 vs. control group by an unpaired Student’s *t* test.

Body weight was evaluated in control and sucrose-fed animals during the 4-week period. Animals fed a high-sucrose diet had increased body weight compared to the control diet (388.5 ± 6.07 g vs. 354.7 ± 9.58 g, *** *p* < 0.001, Figure 1A). Interestingly, 24 h fasting blood glucose levels were unchanged between the two groups (Figure 1B). Food intake was decreased in sucrose fed rats (15.5 ± 1.2 vs. 25.7 ± 0.5 g/day, *** *p* < 0.001; Figure 1C). Sucrose animals had increased water intake compared to the control animals (36.3 ± 1.4 vs. 30.5 ± 0.2 mL/day, ** *p* < 0.01; Figure 1D). The balance between water and food intake resulted in a higher caloric intake in the sucrose fed group (130.2 ± 2.2 vs. 115.5 ± 0.5 cal/day, *** *p* < 0.001; Figure 1E).

### 3.2. Insulin Sensitivity is Impaired in High-Sucrose Diet Fed Animals

Insulin sensitivity was assessed in the control and sucrose groups, in both the fasted and postprandial states. Insulin sensitivity was determined using the rapid insulin sensitivity test (RIST), an adapted hyperinsulinemic and euglycemic clamp [48]. For the control group insulin sensitivity significantly increased from the fasted to the postprandial state, after administration of a commercially available meal (Boost^®^, city, state, country; 103.0 ± 7.2 to 200.4 ± 11.5 mg glucose/kg of body weight, **** *p* < 0.0001; Figure 1F). Animals that were fed a sucrose-enriched diet (35% v/v) have impaired insulin sensitivity, showed by an inability to increase the insulin sensitivity index after administration of the same meal (Figure 1F). Consistent with the observed insulin resistance the sucrose group showed increased circulating levels of both insulin (5.8 ± 0.1 to 40.3 ± 5.6 ng/mL, *** *p* < 0.001; Figure 1G) and c-peptide (3457 ± 788.2 to 6943 ± 922.6 pmol/L, * *p* < 0.05; Figure 1H), pointing a failure in insulin secretion and action. Whole-body glucose homeostasis was further characterized through the meal tolerance test (MTT; Figure 1I). After 4 weeks of high-sucrose feeding, glucose excursion curves were indistinguishable between the control and sucrose-fed animals.

### 3.3. Hepatic Nitric Oxide Expression/Activity and Nitrate Levels, as well as Hepatic Glutathione, are Altered in High-Sucrose Feeding

**Figure 2:**
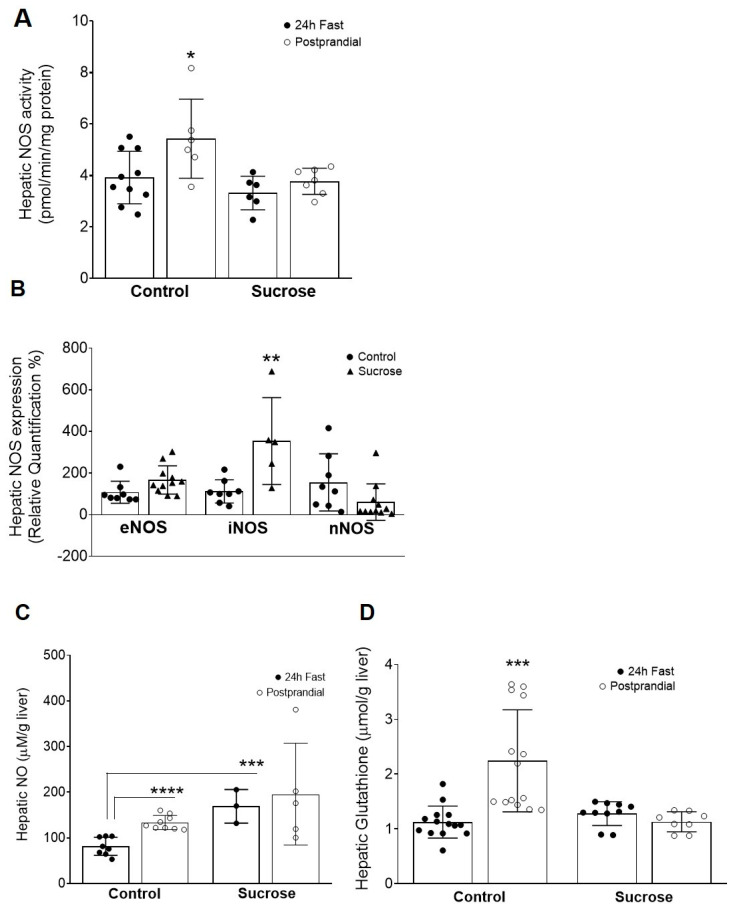
Prolonged exposure to sucrose in the diet leads to changes in hepatic NOS expression and liver levels of NO and GSH. (**A**) Hepatic NOS enzymatic activity in control and sucrose groups; (**B**) gene expression of liver endothelial nitric oxide synthase (eNOS), inducible NOS (iNOS), and neuronal NOS (nNOS) in control and sucrose groups; (**C**) liver levels of nitric oxide (NO) in control and sucrose groups; and (**D**) hepatic glutathione (GSH) in control and sucrose groups. Data are presented as mean ± SEM for 3–11 rats per group. * *p* < 0.05; ** *p* < 0.01; *** *p* < 0.001, and **** *p* < 0.0001 vs. control group by an unpaired Student’s *t* test.

To understand the impact of chronic exposure to dietary sucrose the NOS enzymatic activity was determined. While in the control group we observed a prandial dependent increase in NOS activity this is abrogated in the sucrose group (Figure 2A), showing a negative impact of the high-sucrose feeding on the enzyme’s ability to promote physiological nitric oxide synthesis. Specifically, in the control group there is an increase in the NOS activity from the fast to the postprandial status (3.9 ± 0.3 pmol/min/mg protein vs. 5.4 ± 0.6 pmol/min/mg protein, * *p* < 0.05; Figure 2A), pointing out a role for the constitutive NOS isoforms (endothelial and neuronal) in promoting normal physiological increases in NO production after meal ingestion. Conversely, the sucrose group shows hepatic NOS levels that are unchanged by prandial status, inferring an impairment in the enzyme’s ability to physiologically increase circulating NO in the postprandial status.

Hepatic gene expression of all NOS isoforms was assessed. Neuronal and endothelial isoforms of NOS were unchanged by exposure to sucrose feeding. Interestingly, the inducible isoform of NOS was significantly increased in high-sucrose fed animals (Figure 2B), depicting the activation of iNOS in response to the proinflammatory milieu as a result of chronic exposure to sucrose. This highlights the role of iNOS in promoting a hepatic pro-oxidant niche, a driver of metabolic dysfunction.

Finally, hepatic levels of nitric oxide were further determined. Control animals showed an increase in liver levels of NO, from the fast to the postprandial state (81.6 ± 6.9 vs. 133.4 ± 5.2 µM/g liver, **** *p* < 0.0001, Figure 2C); in contrast, high-sucrose fed animals had increased fasting levels of NO in the liver, compared to control animals (169.1 ± 21.2 vs. 81.6 ± 6.9 µM/g liver, *** *p* < 0.001, Figure 2C) and these were unaffected by feeding (Figure 2C). In summary, the increased hepatic NO levels are due to an upregulation in liver iNOS expression, depicting a role for pro-oxidative NO on the onset of metabolic disorders.

Considering that glutathione was already described as being essential to the increase in insulin sensitivity, seen in the transition from the fast to the postprandial state, hepatic levels of this thiol were determined. In rats fed a standard diet GSH levels increased from the fasting to the fed state (1.12 ± 0.08 vs. 2.2 ± 0.25 μmol/g liver ***, *p* < 0.001; Figure 2D). Rats fed a high-sucrose diet had unchanged liver levels of glutathione despite the prandial status, as was the case for hepatic NO; however, those levels were similar to the ones displayed by control animals in the fast state (1.27 ± 0.07—24 h fast; 1.13 ± 0.06 μmol/g liver—postprandial; Figure 2D). Physiologically, ingestion of a meal promotes liver production of GSH; however, under insulin-resistant conditions, postprandial production of this antioxidant molecule is inhibited rendering these animals prone to hepatic oxidative damage.

### 3.4. Intravenous Administration of S-Nitrosoglutathione Ameliorates Sucrose Induced Insulin Resistance

**Figure 3:**
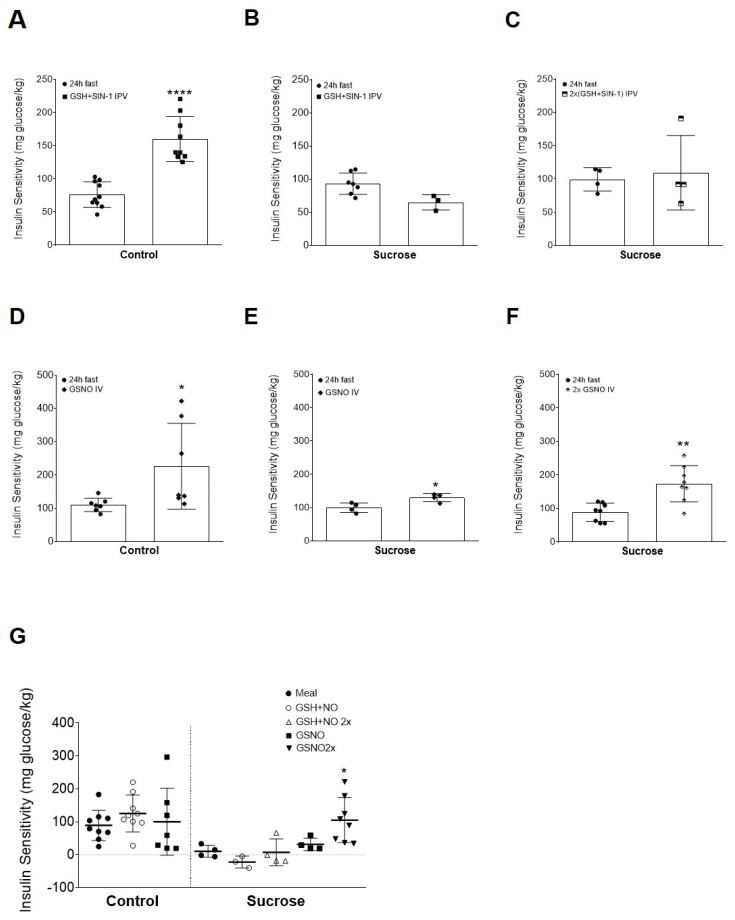
Administration of S-nitrosoglutathione in systemic circulation partially restores sucrose-induced insulin resistance. (**A**) Control group insulin sensitivity after a 24 h fasting period and following administration of a standard dosage of glutathione (GSH—1 mmol/kg of body weight) and SIN-1 (NO donor—50 µmol/kg of body weight), via a portal vein; (**B**) sucrose group insulin sensitivity after a 24 h fasting period and following administration of a standard dosage of GSH and SIN-1, via a portal vein; (**C**) sucrose group insulin sensitivity after a 24 h fasting period and following administration of a dose twice the standard dosage of GSH and SIN-1, via a portal vein; (**D**) control group insulin sensitivity after a 24 h fasting period and following administration of standard dosage of S-nitrosoglutathione (GSNO—50 µmol/kg of body weight), via a femoral vein; (**E**) sucrose group insulin sensitivity after a 24 h fasting period and following administration of standard dosage of GSNO, via a femoral vein; (**F**) sucrose group insulin sensitivity after a 24 h fasting period and following administration of a dose twice the standard dosage of GSNO, via a femoral vein; and (G) summary of the increment in insulin sensitivity, compared to the 24 h fasting period, for both animal groups (control vs. sucrose) and after a meal or drug administration. Data are presented as mean ± SEM for 3–10 rats per group. * *p* < 0.05; ** *p* < 0.01; **** *p* < 0.0001 vs. control group by an unpaired Student’s *t* test.

Previous work had shown that coadministration of both NO and GSH, via a portal vein, was able to mimic a fed-dependent increase in insulin sensitivity [22]. In the control group coadministration of GSH+NO promoted an increase in the insulin sensitivity indexes from the fast to the postprandial state, indicating the importance of GSH and NO for the postprandial insulin sensitivity. The 24 h fasted animals had an insulin sensitivity index of 76.2 ± 6.2 mg glucose/kg of body weight and increased to 159.9±11.4 mg glucose/kg of body weight after 1 mmol/kg GSH and 50 µmol/kg SIN-1 administration (** *p* < 0.01; Figure 3A). However, this effect was not seen in animals fed a high-sucrose diet (Figure 3B). To understand if this impairment was dependent upon the dosage of both molecules a higher dose of GSH and SIN-1 was administered to the sucrose-fed animals. Of GSH 2 mmol/kg and 100 µmol/kg of SIN-1 were not able to significantly change the insulin sensitivity indexes (Figure 3C).

Since the sucrose-fed animals were not sensitive to administration of NO and GSH, it was tested if administration of the physiological end-product of both, into the bloodstream, was able to restore insulin sensitivity. Under physiological conditions NO combines with GSH forming S-nitrosoglutathione (GSNO). Previous work had also shown that intravenous administration of the GSNO was able to mimic the shift in insulin sensitivity seen with changes in the nutritional status. In the standard diet intravenous administration of the GSNO promoted a significant increase in insulin sensitivity. Fasted insulin sensitivity was 110.3 ± 7.6 mg glucose/kg body weight and increased to 226.1 ± 48.7 mg glucose/kg body weight after GSNO administration (* *p* < 0.05; Figure 3D). In the sucrose-fed animals there was a modest increase in insulin sensitivity with administration of the standard GSNO dosage. Insulin sensitivity before S-nitrosoglutathione administration was 100.0 ± 7.3 and increased to 130.1 ± 6.0 mg glucose/kg after GSNO 50 μmol/kg infusion (* *p* < 0.05; Figure 3E). To test if a higher dose was able to further increase insulin sensitivity a second dosage, 100 μmol/kg, was used. A higher GSNO dose was able to significantly increase insulin sensitivity (88.6 ± 9.6 vs. 173.8 ± 19.2 mg glucose/kg), confirming that insulin sensitivity increment was dose-dependent (** *p* < 0.01; Figure 3F).

In Figure 3G the increment in insulin sensitivity for all the groups and drugs administered is summarized. It should be emphasized that the only drug and dosage able to revert the sucrose-induced insulin resistance was the GSNO in a dosage of 100 μmol/kg; however, 50 μmol/kg of GSNO was already modestly but significantly capable of promoting an increase in insulin sensitivity.

### 3.5. S-Nitrosogluathione Promotes Glucose Uptake in L6 Myotubes

In vivo GSNO administration promoted an increase in insulin sensitivity. In vitro experiments were performed to determine if GSNO treatment was, by itself or cotreated with insulin, able to promote glucose uptake in differentiated L6 myotubes.

**Figure 4:**
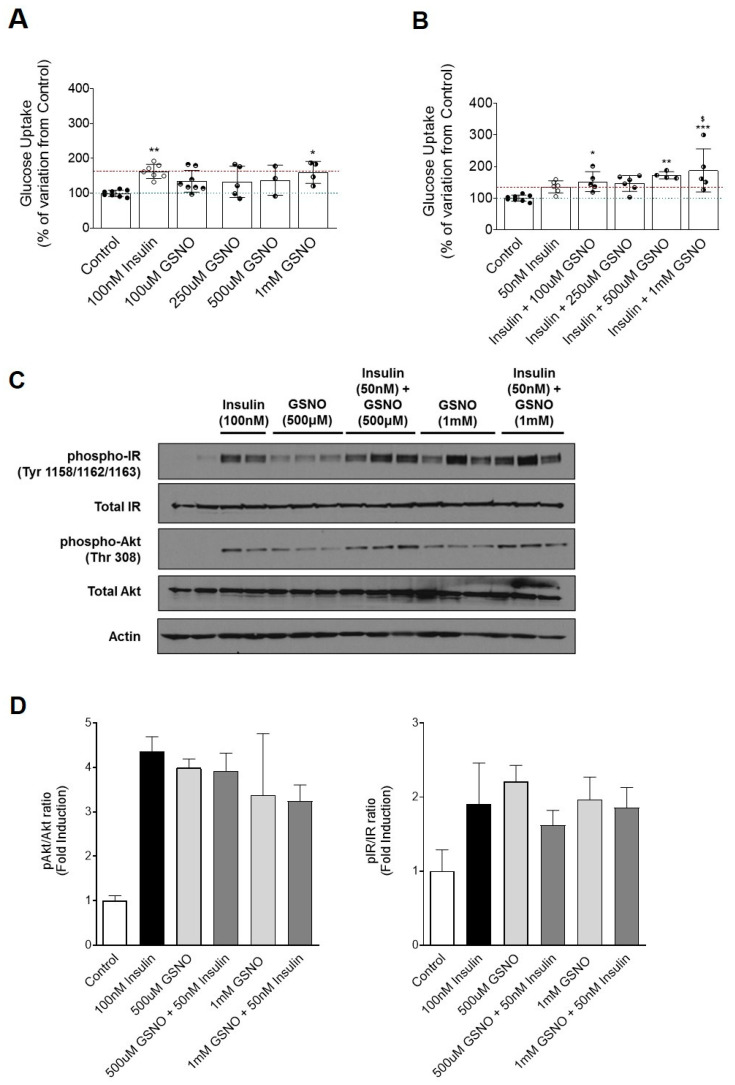
S-nitrosglutathione (GSNO) administration promotes glucose uptake in L6 myotubes and positively modulates the insulin signaling pathway. (**A**) Glucose uptake in L6 myotubes after incubation with insulin (100 nM) or different dosages of GSNO (100–1000 µM); (**B**) glucose uptake in L6 myotubes after incubation with insulin (50 nM) concomitantly with GSNO (100–1000 µM); (**C**) representative images of the phospho- and total insulin receptor (IR) and the phospho- and total Akt (Protein Kinase B) and actin protein levels in L6 myotubes, after stimulation with insulin and/or GSNO; and (**D**) relative quantification of phospho-IR/IR and phosphor-AKT/AKT ratios. Data are presented as mean ± SEM for 3–8 replicates per group. * *p* < 0.05; ** *p* < 0.01, *** *p* < 0.001 vs. control group by a one-way ANOVA; $ *p* < 0.05 vs. 50 nM insulin group by a one-way ANOVA.

When L6 myotubes were stimulated with 100 nM of insulin there was an increase in glucose uptake by 63% (100% ± 3.2% vs. 162.9% ± 7.6%, ** *p* < 0.01, Figure 4A), showing that the cells were properly responding to insulin-induced glucose uptake. When the same cells were treated with GSNO alone there was a maximum increase of glucose uptake for the highest dose of GSNO used, 1 mM (100% ± 3.2% vs. 159.9% ± 15.8%, * *p* < 0.05), suggesting that GSNO may have an insulin-sensitizing effect in muscle cells (Figure 4A).

To further determine if GSNO combined with insulin has a synergistic or additive effect on glucose uptake, L6 myotubes were stimulated with a non-maximal insulin dose (50 nM) and different doses of GSNO, ranging from 100 μM to 1 mM. L6 myotubes stimulation with 50 nM of insulin promoted an increase in glucose uptake by 35%. Treatment of muscle cells with GSNO and insulin induced an increase in glucose uptake by 90%, at a concentration of 1 mM (100.0% ± 3.4% vs. 187.6% ± 30.4%, *** *p* < 0.001 and *p* < 0.05 vs. 50 nM of insulin; Figure 4B).

Taken together, these data, combined with the previous in vivo data, indicate that GSNO is able to promote glucose uptake in muscle cells. When combined with insulin it has an additive effect on whole-body glucose homeostasis and insulin sensitivity.

### 3.6. S-Nitrosoglutathione Promote Glucose Uptake by Activating the Insulin Signaling Pathway

To determine the mechanism by which GSNO regulates glucose uptake in muscle cells, the proximal insulin signaling components were assessed. As expected, insulin greatly increased IR and Akt phosphorylation in L6 muscle cells. Importantly, treatment of muscle cells with GSNO also led to a significant increase in IR and Akt phosphorylation (Figure 4C,D). Similar observations were detected when treating with GSNO combined with insulin (Figure 4C,D). These data suggest that GSNO-mediated improvement of insulin sensitivity is associated with enhanced insulin signaling.

## 4. Discussion

The data presented in this study shows for the first time that administration of GSNO is sufficient to overcome high sucrose-induced insulin resistance in rats. This effect is associated with a positive modulation of the insulin signaling pathway. We also found that both nitric oxide and glutathione are fundamental for maintaining normal insulin action, as their levels are impaired in the early stages of insulin resistance. However, administration of both molecules was unable to restore insulin action in the high sucrose rat model. Rather, peripheral administration of the end product of glutathione and nitric oxide combined, GSNO, was effective in restoring insulin sensitivity. A chronic sucrose-induced pro-oxidant environment leads to a defect in the GSNO production, thus impacting on its role as an insulin-sensitizing molecule. Finally, an in vitro study using L6 myotubes emphasizes the importance of GSNO as a modulator of glucose metabolism, as revealed by the evidence that stimulation of these cells with GSNO increased glucose uptake and insulin signaling at the level of the insulin receptor and Akt.

### 4.1. Body Weight, Water/Food Intake, and Biochemical Parameters

Animals on a high-sucrose diet had increased body weight when compared to standard animals but increased daily caloric intake, showing that increased sugar consumption leads to higher body weight. Several studies show a positive correlation between body weight and sugar intake; although different outcomes may arise depending on a number of factors, including study designs, liquid versus solid diet, type of sugars studied, etc. [49,50,51,52]. Our data is in accordance with what has already been described for Wistar rats under a high-sucrose diet for a 4 week period, being that this is long enough of a period to significantly impair insulin action [53]. This was observed in our work by an inability of high-sucrose fed animals to properly respond to meal ingestion, showing postprandial insulin resistance. However, it is important to highlight that this impairment in insulin action is dependent on the nutritional status as insulin action was normal in the fasting state.

### 4.2. Weeks of Sucrose-Enriched Diet Leads to Impaired Insulin Secretion and Clearance in the Fed State

The data presented herein shows that 4 weeks of sucrose intake with drinking water does not lead to hyperglycemia during the meal tolerance test. This observation is consistent with the previous findings [54]. In fact, Pinyo et al. have reported that 4 weeks of high-sucrose feeding did not significantly alter glucose excursions, after ingestion of a meal; however, they observed a marked increase in insulin levels, during the MTT, which can account for normal glycemia. It is expected that in our animal model of high-sucrose feeding circulating levels of both insulin and c-peptide would be upregulated, as a pancreatic compensatory mechanism to prevent hyperglycemia and maintain glucose homeostasis. Martins et al. have also observed that with a 2 week period of high-sucrose feeding (35% in drinking water) there was no impact on blood glucose levels, after ingestion of an identical meal to the one used in our studies; nevertheless, the authors have showed that insulin levels were increased during the MTT, again showing that the pancreatic β-cell is partially responsible for unchanged glycemia [55]. These observations can be translated into the clinic, where it has been observed that although apparently healthy individuals show no changes in a glucose tolerance test, they may already have impaired insulin action [56].

### 4.3. Glutathione and NO Levels are Impaired after 4 Weeks of Sucrose Diet

It is evident that impaired NO plays a role in insulin resistance and T2D in humans [57,58,59] and the negative impact of hypercaloric diets on GSH levels is documented, stressing the importance of GSH action to maintain a proper antioxidant system and whole-body homeostasis [60,61]. Here we show that dietary sucrose led to an increase in iNOS expression, which can account for the increase in hepatic NO. These observations are in accordance with what has been recently discovered for iNOS. Under inflammatory and oxidative stress environments, iNOS-derived NO seems to be a modulator of numerous biochemical pathways and energy metabolism, namely glucose and lipid metabolism [62].

Blouet et al. concluded that 4 weeks in a high sucrose diet promotes an increase in nitrotyrosine plasma levels, which is in accordance with the overall inflammatory process [20]. These authors tried to sustain the reactive nitrogen species (RNS) production by administration of cysteine to high sucrose fed animals. Administration of cysteine was able to decrease the levels of RNS but insulin resistance persisted. We have also observed that the hepatic iNOS expression was increased in this model, which is in accordance with results obtained by Blouet et al., highlighting that the inflammatory process is one of the major alterations occurring on the onset of diet-induced insulin resistance. Finally, it is important to note that an increase in NO levels may lead to hyperinsulinemia, by suppressing insulin clearance, a process that was previously observed in humans by Natali et al. [58].

Francini et al. also observed that the hepatic antioxidant system was decreased by a fructose-rich diet. This effect was accompanied by decreases in GSH and catalase levels [19]. Supporting this, Du et al. also found that high sugar diets induced oxidative stress by decreasing hepatic expression of the major enzymes involved in the antioxidant defense system [18]. In this study, we observed that hepatic GSH content was decreased, mainly in the fed state.

Taken together, these data show that the normal physiological reaction between NO and GSH is compromised by chronic exposure to sucrose in the diet. Thus, the inability to generate the S-nitrosoglutathione from these molecules could be a crucial factor in promoting insulin resistance.

### 4.4. S-Nitrothiols are Sufficient to Overcome Insulin Resistance

Since our previous work showed the importance of hepatic glutathione and nitric oxide on proper insulin action, it is hypothesized that an intraportal vein administration of both would be sufficient to overcome dietary sucrose induced-insulin resistance [22,23]. In contrast to our hypothesis, we found that coadministration of GSH and NO to the liver had no effect on insulin sensitivity. This could be due to a lack of physiological reaction between GSH and NO, which could lead to impaired bioavailability of these agents in improving insulin action. Future studies are needed to address this critical issue.

However, we have shown, for the first time, that systemic administration of GSNO is able to restore insulin action, compromised due to the exposure to a high sucrose diet. GSNO is part of the larger group of S-nitrosothiols, which have recently emerged as important modulators of cell function, due to their capacity to nitrosylate proteins, activating or inactivating their function [26,29,63,64]. Previous work from our group had already shown the importance of intravenous administration of GSNO in promoting an increase in insulin sensitivity in lean healthy Wistar rats [32].

Several works reported these drugs as important mediators of the insulin signaling pathway by increasing GLUT4 translocation to the plasma membrane, as evidenced by the findings that S-nitroso-N-acetilpenicilamine treatment in skeletal muscle cells increased the GLUT4 expression via AMPK [34]. This is further supported by the findings that NO enhanced insulin-mediated glucose uptake in skeletal muscle strips of both normoglycemic and type 2 diabetic rats [64].

Our in vitro results further reinforce the role of GSNO as a modulator of insulin action. Stimulation of myotubes with GSNO led to an increase in glucose uptake, especially when this is coadministered with insulin. Additionally, this increase in insulin stimulated glucose uptake was accompanied by an increase in phosphorylation of key mediators of the insulin signaling pathway. This indicates a possible mechanism for GSNO effect in overall glucose metabolism through activation of insulin signaling, namely the insulin receptor and protein kinase B/Akt. Indeed, a study published by de Castro Barbosa et al. had already shown that administration of L-arginine, the physiological precursor of NO, positively impacted glucose and lipid metabolism, via phosphorylation of Akt [33]. Along with this, we also showed, in an animal model of insulin resistance, that these drugs could overcome dietary sucrose-induced insulin resistance indicating GSNO as a potential pharmacological tool.

## 5. Conclusions

**Figure 5:**
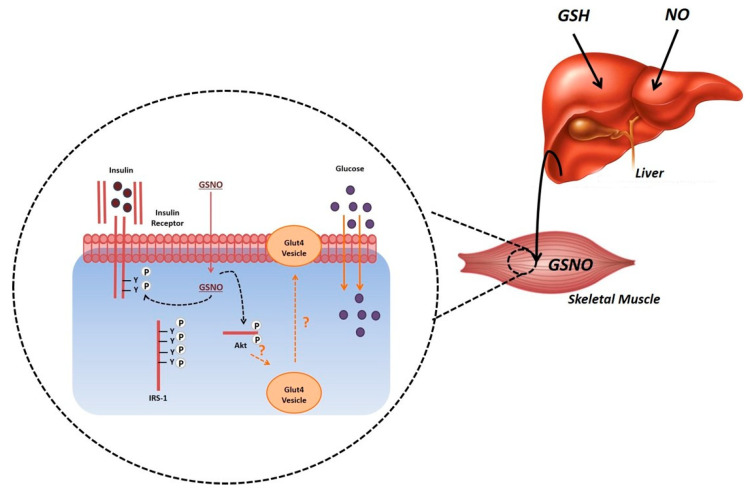
Hepatic production of S-nitrosoglutathione may positively impact insulin sensitive tissues through modulation of the insulin signaling pathway. Abbreviations: GSH—glutathione, GSNO—S-nitrosoglutathione, NO—nitric oxide.

In conclusion, dietary sucrose-induced insulin resistance leads to impaired hepatic metabolism and antioxidant capacity. This defect is rescued by improving insulin’s metabolic action when rats are treated with GSNO. Thus, we propose a metabolic model for S-nitrosoglutathione action as an insulin sensitizer. This molecule interacts with insulin forming a molecular complex that could bind to the IR activating the downstream signaling cascade, leading to the translocation of the GLUT4 vesicles to the plasma membrane. As a result, glucose uptake in the muscle is increased (Figure 5). Finally, our findings suggest a crucial role for glutathione and nitric oxide in regulating glucose metabolism that cannot be overlooked as it can represent an alternative for the treatment of insulin resistance in obesity and type 2 diabetes.
